# Nitrogen-to-Protein Conversion Factors for Edible Insects on the Swiss Market: *T. molitor, A. domesticus*, and *L. migratoria*

**DOI:** 10.3389/fnut.2020.00089

**Published:** 2020-07-10

**Authors:** Samy Boulos, Anina Tännler, Laura Nyström

**Affiliations:** Laboratory of Food Biochemistry, Department of Health Sciences and Technology, Institute of Food, Nutrition and Health, ETH Zurich, Zürich, Switzerland

**Keywords:** edible insects, nitrogen-to-protein conversion, amino acid profile, chitin, protein overestimation, amide nitrogen, protein recovery, true protein

## Abstract

With an increasing worldwide demand for animal protein, insects are becoming a promising sustainable option for meat protein replacement. However, reported protein contents of insects are often overestimated when calculated as “crude protein” = 6.25 × nitrogen content (*N*), compared to true protein contents quantified from the sum of amino acid (AA) residues. In this study, the main two types of usual nitrogen-to-protein conversion factors *k*_*p*_ and *k*_*A*_ were determined on the basis of true protein/total nitrogen and true protein/protein nitrogen, respectively, with focus on the three insect species legally sold on the Swiss food market. *T. molitor* (mealworm larvae), *A. domesticus* (house crickets), and *L. migratoria* (locusts) from various breeders were analyzed for total and amide nitrogen, chitin, and AA composition. Careful control experiments of insect samples spiked with a protein standard were conducted to establish the recovery of true protein, which was with >95% excellent. Mealworms, crickets, and locusts exhibited similar AA-profiles and true protein contents of 51, 55, and 47 g/100 g (dry weight basis), respectively. Specific conversion factors *k*_*p*_ showed little variability between the three insect species with 5.41, 5.25, and 5.33 for mealworms, crickets, and locusts, respectively, and confirmed an average ~17% overestimation of protein contents when using 6.25 × *N*. The determined average *k*_*p*_ of 5.33 is supported by extracted literature data and is suggested for general use instead of 6.25 × *N* to calculate more accurate insect protein contents, whereas the average pure protein conversion factor *k*_*A*_ of 5.6 is proposed for use in the case of insect protein isolates.

## Introduction

The worldwide demand for animal protein increased over the past years and is presumed to continue to increase by over 50% until 2050, mainly driven by the population growth and increasing wealth. However, the land requirements for animal forage crop production will exceed the available areas on the globe in the future. Hence, alternative protein sources are needed to meet the predicted demand. The high protein content with the presence of all essential amino acids (AA) and the ease of rearing make insects a promising option for meat protein replacement (1–3).

While insects are already consumed in many parts of the world, a new food law was only recently introduced to Switzerland in May 2017 which allows three insect species, namely *Tenebrio molitor* (yellow mealworms), *Acheta domesticus* (crickets), and *Locusta migratoria* (locusts), to be bred and sold as food products without requiring a permission (4).

The protein content in food is often determined on the basis of the total nitrogen content. Kjeldahl or elemental analysis methods are almost universally applied for nitrogen determination. The protein content is then calculated by multiplying the nitrogen content with the nitrogen-to-protein conversion factor (*k*_*p*_). Most frequently, factors published by Jones (5) are used, who proposed as factor for animal proteins and as default factor for unknown proteins the value 6.25. This corresponds to an average nitrogen content of 16% in the pure protein (100%: 16% = 6.25) (6). Even though Jones' values have been shown to be erroneous due to (1) the applied extrapolation of the nitrogen content from isolated protein fractions of unreported purity to the whole tissue, and (2) negligence of the non-protein nitrogen fraction, they are still used in food composition tables (7). Based on actual conversion factors for proteins from plant and animal sources, Mariotti et al. proposed a new default average factor of *k*_*p*_ = 5.6. Nevertheless, as for many other food proteins, it is still general practice to apply *N* × 6.25 to calculate “crude protein” contents of insects, as conversion factors for insects are scarce. Consequently, the high levels of protein contents reported in insects are often overestimated (8). It is not conclusively settled if a (partial) correction is possible by subtracting the non-protein nitrogen contained in the chitin exoskeleton from the total nitrogen content before calculating crude protein by *N* × 6.25. Hence, amino acid analysis is the only method to give reliable, accurate results for protein contents (9), and if not possible, use of an accurate, specific conversion factor for the food item is suggested (6).

To the best of our knowledge, only Janssen et al. (10) and Belghit et al. (11) published specific conversion factors for some insect species, including a *k*_*p*_ of on average around 4.7 for both yellow mealworms and crickets. Conversion factors for locusts, on the other hand, are yet to be determined. The other conversion factor type *k*_*A*_ [9], which is of practical relevance to calculate the protein content for protein isolates, have together with the required amide nitrogen (*N*_*amide*_) content not yet been reported for insects. What comes closest is a *k*_*p*_ calculated for a mealworm protein extract of 5.6 from Janssen et al. (10), but none for crickets or locusts. Hence, the overall aim of this study was the determination of accurate, insect-specific nitrogen-to-protein conversion factors (*k*_*p*_ and *k*_*A*_) and the evaluation to which extent chitin nitrogen is responsible for the protein overestimation when using the universally applied *k*_*p*_ = 6.25. Analyses were performed with three edible insect species which are commercially sold in Switzerland, namely *T. molitor* (yellow mealworm), *A. domesticus* (house crickets), and *L. migratoria* (locusts) by measuring the contents of amino acids, nitrogen and chitin. The conversion factors and composition, as well as the quality of the proteins were evaluated and compared with regard to the three insect species and the different breeders.

## Materials and Methods

### Chemicals

α-Methyl-DL-tryptophan (α-Me-DL-Trp, crystalline), L-tryptophan (Trp, 99.5%), amino acid analytical standard, L-norleucine (NorLeu, 98%), and phenyl isothiocyanate (PITC, ≥99%) as well as all other solvents and chemicals were of analytical quality and were purchased from Sigma-Aldrich (Buchs, Switzerland). Bovine serum albumin (BSA; 97%), was bought from VWR International GmbH (Dietikon, Switzerland). Water of Milli-Q quality (Merck Millipore) from Merck KGaA (Darmstadt, Germany) was used for all experiments. All mixtures of liquids given as ratios refer to volume to volume ratios (v/v) unless otherwise noted.

### Insect Samples

Insect samples were kindly provided by Essento Food AG, Switzerland for the analysis. *T. molitor* larvae (mealworm) samples originated from three different breeders (company name in parenthesis), namely M1 (Essento), M2 (Entomos AG, Switzerland), and two different batches from the same third breeder, M3a and M3b (Bugood Food SPRL, Belgium). *A. domesticus* (house cricket) batches came from the two breeders, C1 (Little Food, Belgium) and C2 (same breeder as M2, namely Entomos AG), and *L. migratoria* (locusts) from one breeder L1 (Pollner Insektenzucht, Austria). All insects were food grade and supplied frozen at −20°C. According to the provider, the insects underwent a starving period of 24 h before being harvested, frozen, thawed, blanched, cooled down and stored at <−20°C. Wings and legs (w+l) of locusts were removed and analyzed separately from the rest of the body, as they are usually not consumed unless further processed. Presented locust values for the whole insect are calculated by taking the weighted averages of the two components' results according to their respective proportion of the whole insect (87.3% body, 12.7% w+l on dwb). Frozen insects were lyophilized (Lyolab Bii, LSL Secfroid, Aclens, Switzerland/ vacuum pump Trivac, Leybold-Haraeus, Switzerland) in batches of 50–80 g for 3 days at −50°C and the exact weight loss recorded. Each dried insect batch was ground to a fine powder with a mill (Grindomix GM200, Retsch GmbH, Haan, Germany) using 5 × 10 s pulses at 10,000 rpm and stored at −20°C until analysis.

To determine the residual moisture, ~300 mg of each freeze-dried insect powder was dried in an oven in triplicates at 100°C for 48 h (determined as sufficient time by pre-test heating until constant weight) and cooled down in a desiccator. The residual moisture content was calculated from the observed weight loss. All presented contents of amino acids (AA), nitrogen, protein, chitin, and glycogen are on dry weight basis (dwb), and [g/100 g] refers to [g/100 g insect powder (dwb)] if not explicitly stated otherwise.

### Total Nitrogen and Amide Nitrogen Contents

The total nitrogen content (*N*_*total*_) in insects was measured with an organic elemental analyzer (FlashEA 1112 Series, Thermo Fisher Scientific, Schlieren, Switzerland). Approximately 1 mg of each insect powder was weighed in quadruplicates into a tin foil capsule (Säntis Analytical, Teufen, Switzerland). The nitrogen content of samples with relative standard deviations (RSD) larger than 8% due to powder inhomogeneity, namely the locust body sample, was measured in quadruplicates with the standard Kjeldahl method (250 mg per replicate) (12). This confirmed the average nitrogen content, but with smaller RSD.

The amide nitrogen content (*N*_amide_) corresponding to nitrogen in the AA side chains (R) of Asn + Gln (R = –(CH_2_)_x_CONH_2_, with x = 1 or 2, respectively) was determined according to Mossé et al. (13), using a milder hydrolysis method (compared to AA analysis) to liberate amide ammonia (X–CONH_2_ + H_2_O → X–COOH + NH_3_). Briefly, ~300–1,000 mg of insect sample (depending on insect) was weighed accurately into 30 mL pyrex tubes in triplicate, and 20 mL 2 M HCl added together with a magnetic stirring bar and the tubes sealed. The samples were incubated at 115°C in a heating block (Reacti-Therm III, Thermo Fisher Scientific, Schlieren, Switzerland) under stirring for 3 h, the tubes then removed and cooled to room temperature. The content was quantitatively transferred to Kjeldahl flasks with the help of in total 30 mL H_2_O, and put into the steam distillation unit B-324 (BÜCHI Labortechnik AG, Flawil, Switzerland) with connected water- and 32% (w/w) NaOH-tanks. The automatic steam distillation was programmed to add 30 mL NaOH solution, followed by distillation with 100% steam for 4 min, collecting ~100 mL distillate into 30 mL of 40 g/L boric acid solution. The captured ammonia in the boric acid solution was then titrated with 0.025 M HCl until color change of the added Tashiro indicator (green to pink) and the amide nitrogen content in [g/100 g insect] calculated:

(1) $$\begin{array}{l} N_{amide}\left[\frac{\text{g}}{100\text{ g }}\right]=\frac{titration\;volume\;\left[\text{mL}\right]\;\times \;0.35017\;\left[\frac{\text{mg}}{\text{mL}}\right]}{sample\;mass\;\left[\text{mg}\right]}\\ \;\times 100\left[\frac{\text{g}}{100\text{ g }}\right]. \end{array}$$

In addition, control experiments with each 50 mg glutamine and asparagine were conducted, as well as 50 mg chitosan and arginine. They all confirmed high recovery for Asn and Gln amide nitrogen, and allowed for the correction of the *N*_*amide*_ results through the minimal contamination from arginine and chitin decomposition. The degree of amidation was then calculated using molar amounts of detected *N*_*amide*_, Asx, and Glx (representing the sum of Asn+Asp and Gln+Glu, respectively; see section Analysis of Amino Acid Composition) by

(2) $$\begin{array}{l} \text{degree of amidation }\left[\text{\%}\right]=\frac{N_{amide}^{\left[\text{mol}\right]}}{{\left(\text{Asx}+\text{Glx}\right)}_{\left[\text{mol}\right]}}\times 100\%. \end{array}$$

*N*_*amide*_ also allows for the calculation of the amount of nitrogen coming from protein (*N*_*protein*_), which is the total nitrogen recovered from the 18 amino acid residues (AAR) + *N*_*amide*_ from amide side chains,

(3) $$\begin{array}{l} N_{protein}\left[\frac{\text{g}}{100\text{ g }}\right]=\overset{18}{\underset{\text{i}=1}{\sum }}\left({\text{AAR}}_{i}\left[\frac{\text{g}}{100\text{ g }}\right]\times \frac{n_{i}\;\times \;14.007\;\left[\frac{\text{g}}{\text{mol}}\right]}{M_{w}\left({\text{AAR}}_{i}\right)\left[\frac{\text{g}}{\text{mol}}\right]}\right)\\ \;+\;N_{amide}\left[\frac{\text{g}}{100\text{ g }}\right] \end{array}$$

with AAR_*i*_ being the *i*th anhydrous amino acid residue content in the insect, *n*_*i*_ = number of nitrogen atoms of the *i*th AAR molecule, 14.007 [g/mol] corresponding to the molecular weight (*M*_*w*_) of nitrogen, and *M*_*w*_(AAR_*i*_) = *M*_*w*_(AA_*i*_) – *M*_*w*_(H_2_O) = *M*_*w*_ of the anhydrous amino acid residue.

### Analysis of Amino Acid Composition

The amino acid (AA) composition of freeze-dried insect powder was determined (at least) in triplicates using phenylisothiocyanate (PITC) derivatization according to Kwanyuen and Burton (14) and White et al. (15), with minor modifications in the hydrolysis procedure.

#### Hydrolysis and Precolumn Derivatization

Lyophilized insect powder (30 mg) was placed into a 10 mL pyrex tube. Then, 2 mL of 6 M HCl containing 0.1% phenol (m/v) and 1 mL of 6 M HCl containing 1 mg of NorLeu (as internal standard) were added. The pyrex tube was flushed with nitrogen and incubated in a heating block (Reacti-Therm III, Thermo Fisher Scientific, Schlieren, Switzerland) under stirring at 110°C for 24 h (confirmed as optimal time point to maximize recovered amino acids). The hydrolyzed sample was cooled down to room temperature, filtered (0.45 μm), and a 30 μL aliquot vacuum dried (SpeedVac Savant, Thermo Fisher Scientific, Switzerland) at 35°C. Then, the dried sample was neutralized with 30 μL 2:2:1 EtOH/ H_2_O/ NEt_3_, vortex mixed and vacuum dried again. The derivatization was performed by the addition of 60 μL 7:1:1:1 EtOH/ H_2_O/ NEt_3_/ PITC (freshly prepared) to the dried and neutralized sample. After 20 min incubation at room temperature, the sample containing the formed phenylthiocarbamyl (PTC) amino acids was dried under vacuum and stored at −20°C until analysis.

#### RP-HPLC Analysis

The dried PTC amino acid pellet from the sample or standard was dissolved in 1.5 mL of a 5 mM Na_2_HPO_4_ buffer (pH 7.4; containing 5% ACN), filtered (0.45 μm), and 10 μL injected into an RP-HPLC Agilent 1100 series LC-system (Agilent Technologies, Santa Clara, USA). For the separation of the molecules, a C18 analytical Pico-Tag amino acid analysis column (3.9 × 150 mm) was used in combination with a Nova-Pak C18 guard column (3.9 × 20 mm) (Waters AG, Baden, Switzerland). The PTC amino acids were separated by a gradient of eluent A (150 mM sodium acetate, 0.05% NEt_3_ and 6% ACN, adjusted to pH 6.4 with 10% acetic acid) and eluent B (6:4 acetonitrile/ water). The flow rate was set to 1 mL/min, the temperature of the column was maintained at 38°C, and the total run time was 23 min with the following gradient profile: 100% A at the beginning, going down to 80% A and 20% B within 5.5 min, and linearly to 54% A and 46% B at 10 min, changing to 100% B until 10.5 min and staying there until the 12th min, then switching back to 100% A until the 13th min, followed by 10 min re-equilibration with eluent A. The eluted PTC amino acids were detected at 254 nm by a UV/Vis-diode array detector (for chromatogram examples, see Figure S1 in the Supplementary Material).

The calibration curve was constructed as follows: To 1 mL of Sigma Aldrich AA standard mixture (2.5 μmol/mL for each AA in 0.1 M HCl, except for cysteine with 1.25 μmol/mL), 250 μL of a 10 mM NorLeu solution in 0.1 M HCl was added to reach a final concentration of 2 μmol/mL for each AA (cysteine: 1 μmol/mL). Specific amounts of 7.5–75 μL of the standard mixture were dried under vacuum, neutralized, derivatized with PITC in duplicates, dried, and dissolved in 1.5 mL Na_2_HPO_4_ buffer as described above under sections Hydrolysis and Precolumn Derivatization and RP-HPLC Analysis, generating standard solutions of 10–100 μM (100–1000 pmol per 10 μL injection). The determined AA amounts [pmol] for the samples were divided by each injection's internal standard recovery [%] (NorLeu) to correct for losses during sample preparation. Cysteine (Cys) was additionally corrected by multiplication with a factor as determined in section Hydrolysis Time Pre-Tests and BSA-Spiking.

#### Calculation of True Protein and Conversion Factors

The corrected AA amounts in [pmol] were converted to mass contents [g/100 g insect] by using the AA's anhydrous residue molecular weight (*M*_*w*_) representing the AA in the peptide chain of the protein, meaning *M*_*w*_(AAR) = *M*_*w*_(AA)–*M*_*w*_(H_2_O) (minus 18 g/mol). True protein was then calculated as the sum of the 18 quantified anhydrous amino acid residue amounts (AAR_*i*_) (incl. Trp from section Tryptophan Analysis):

(4) $$\begin{array}{l} true\;protein\;\left[\frac{\text{g}}{100\;g\;}\right]=\overset{18}{\underset{i=1}{\sum }}\left(AAR_{\text{i}}\left[\frac{\text{g}}{100\;g\;}\right]\right) \end{array}$$

These 18 represent all 20 biogenic AA, as detected Asp and Glu represent the amounts of Asn+Asp (=Asx) and Gln+Glu (= Glx) in the protein, respectively. AA profiles are reported for each AA residue relative to the true protein content in [g/100 g protein]. Conversion factors *k*_*p*_ and *k*_*A*_ were then calculated according to the following two equations:

(5) $$\begin{array}{l} k_{p}=true\;protein\;\left[\frac{\text{g}}{100\text{ g }}\right]\;/\;N_{total}\left[\frac{\text{g}}{100\text{ g }}\right] \end{array}$$

(6) $$\begin{array}{l} k_{A}=true\;protein\;\left[\frac{\text{g}}{100\text{ g }}\right]\;/\;N_{protein}\left[\frac{\text{g}}{100\text{ g }}\right]. \end{array}$$

#### Hydrolysis Time Pre-tests and BSA-Spiking

To ensure that 24 h is sufficient time for the hydrolysis, a sample of each insect type as well as pure BSA was incubated for 24 and 48 h and the relative AA profiles and total protein contents (24 vs. 48 h) compared. Additionally, to observe any potential AA degradation during the hydrolysis that is not sufficiently corrected by the NorLeu internal standard, 10 mg of bovine serum albumin (BSA) with and without insect powder were hydrolyzed (for 24 h) and prepared with the same procedure as described above (section Hydrolysis and Precolumn Derivatization and RP-HPLC Analysis). The experimental AA profile of BSA was in agreement with literature values (16) and no statistically significant degradation of AA due to the insect matrix was found, except for cysteine (average AA recovery of 98%). Based on the spiking results with BSA, a cysteine correction factor of 2.5 was applied to reach a fully recovered amount of total cysteine in the insect samples (17, 18). In addition, BSA total protein recovery proved to be with >95% excellent for both BSA alone and BSA with insect powder.

### Tryptophan Analysis

The hydrolysis for quantifying tryptophan (Trp) was performed in quadruplicates under basic conditions with sodium hydroxide and analyzed by RP-HPLC in combination with a fluorescence detector according to Çevikkalp et al. (19) and Zhang et al. (20).

#### Sample Preparation

Sixty milligram of lyophilized insect powder was put into a 10 mL pyrex tube, followed by 2 mL of 5 M NaOH and 1 mL of 5 M NaOH containing 0.5 mg α-methyl-DL-tryptophan (α-Me-Trp) as internal standard. The pyrex tube was flushed with nitrogen and incubated under stirring in a heating block at 110°C for 20 h. The hydrolysate was cooled down to room temperature, the pH adjusted to 6.3 by the addition of HCl, and the solution was quantitatively transferred and filled up to 100 mL in a volumetric flask. The diluted sample was centrifuged for 5 min at 4,000 rpm (Eppendorf 5810R, Vaudaux-Eppendorf, Basel, Switzerland). The supernatant was diluted three times with water, filtered (0.45 μm), and 10 μL injected into the HPLC-system.

#### HPLC Analysis

The same HPLC system and column was used as for the measurement of the other AA (see RP-HPLC Analysis), but in combination with a fluorescence detector. The mobile phase was composed of 25 mM sodium acetate buffer with 10% ACN. The flow rate was set at 1 mL/min and the column maintained at 30°C with a total run time of 10 min, resulting in a retention time of 2.0 min for Trp. The fluorescence detector was operated at an excitation wavelength of 280 nm and an emission wavelength of 340 nm. 10 μL of 1–50 μM Trp and α-Me-DL-Trp standards were injected in duplicates for the generation of a calibration curve. The detected amounts of Trp in the samples were corrected relative to the α-Me-Trp recovery to account for losses during sample preparation (see Figure S2 for a chromatogram example).

### Chitin and Glycogen

The chitin and glycogen content of freeze-dried insect powder was determined in quadruplicates after complete hydrolysis by high-performance anion-exchange chromatography with pulsed amperometric detection (HPAEC-PAD).

#### Chitin Hydrolysis

The hydrolysis of chitin and glycogen into their monomers glucosamine (GlcN) and glucose (Glc) was done according to Gilbert-López et al. (21) and Saeman et al. (22). 60 mg of freeze-dried insect powder was placed into a 10 mL pyrex tube. Then, 0.5 mL of 72% (w/w) sulfuric acid was added and kept at 30°C for 1 h in a heating block under stirring, followed by 5.5 mL water to result in 1 M H_2_SO_4_, and the tube incubated at 100°C for 4 h. The hydrolyzed sample was cooled down to room temperature and centrifuged at 4000 rpm for 10 min. Exactly 125 μL of the supernatant was neutralized with 250 μL 1 M NaOH to reach pH 6–8 and filled up to 5 mL with water. The diluted sample was vortex mixed, filtered (0.45 μm), and 10 μL injected into the HPAEC-PAD system (see Figure S3 for a chromatogram example).

#### HPAEC-PAD Analysis

A Dionex ICS 5000 chromatography HPLC system (Thermo Fisher Scientific, Switzerland) equipped with a CarboPac PA1 column (2 × 250 mm) in combination with a CarboPac PA1 guard column (2 × 25 mm) and a pulsed electrochemical detector in pulsed amperometric detection mode was used for the measurement of the sugar monomers according to a published method (23). The temperature of the column was set at 25°C with a flow rate of 1 mL/min. The monosaccharides were separated by a gradient using MilliQ water for eluent A and 200 mM NaOH solution for eluent B. The 37 min run had the following elution profile: 92% A and 8% B isocratically for the first 20 min, 100% B between 20.5 and 29 min, 92% A and 8% B at 29.5 min until the end. GlcN-HCl and Glc solutions with concentrations between 5 and 100 μM were used for the calibration, whereof 10 μL were injected into the system. The reported chitin and glycogen values are given on the basis of anhydrous monomer residues to represent the actual polysaccharide contents in the insects.

### Statistical Analysis

ANOVA was performed to compare the mean group values with a significance level of 0.05 by the IBM SPSS statistic program (IBM Schweiz AG, Zurich, Switzerland). Tukey HSD was used if the data had a homogenous variance and same number of replicates for all groups. Hochberg's G2T was used when sample sizes of groups were unequal and Games Howell was performed when homogeneity of variance was not given. Standard deviations of averages calculated from other averages that already have a standard deviation (e.g., averaged AA composition of the three insect species) were calculated taking the quadrature of the internal (*SD*_*int*_) and external standard deviation (*SD*_*ext*_) [see equations (S1–S5) in the Supplementary Material; (24)].

## Results and Discussion

### Protein, Nitrogen and Chitin Composition

The true protein content, based on the sum of anhydrous amino acid residues (Σ[AAR_*i*_]), showed for crickets with an average of 54.9 g/100 g (dry weight basis; dwb) the highest amount, followed by mealworms and locusts with 50.9 and 46.6 g/100 g, respectively (see Table 1). In the literature, most reported protein contents of insects are “crude protein” based on 6.25 × *N*_*total*_, even when amino acid (AA) analyses where done. Crude protein, which does not distinguish between protein nitrogen and non-protein nitrogen, is usually expected to be higher than true protein from AA analysis. Interestingly, our observed true protein contents of mealworms and locusts were comparable with crude protein values reported in the literature, whereas for crickets, literature values were with 60–70 g/100 g crude protein as expected higher than our value of 54.9 g/100 g true protein, namely by up to a quarter (25–31). Only a few papers either give true protein contents directly or report enough information to allow the calculation of Σ[AAR_*i*_] to be able to compare their true protein (dwb) to our results. These directly or indirectly reported values are in the range of 37–52 g/100 g (dwb) for mealworms, with our mentioned average of 50.9 g/100 g being at the upper end of the range (10, 11, 25, 27, 32). With crickets, on the other hand, true protein ranges in the literature between 41–53 g/100 g, this time slighty below our observed average of 54.9 g/100 g (10, 11, 27, 32, 33).

**Table 1:** Averages of amino acid (AA) composition [g/100 g true protein] and contents of true protein, chitin, glycogen, and nitrogen [g/100 g insect, dwb] are listed for mealworms, crickets, and locusts, as well as nitrogen–to–protein conversion factors (*k*_*A*_ *and k*_*p*_) and averages of all three insect species^a^.

		***T. molitor* (mealworms)**	***A. domesticus* (crickets)**	***L. migratoria* (locusts)**	**Average of the three insects**
Amino acid residue (AAR) [g/100 g true protein]	*His	4.2 (±0.3)	3.1 (±0.3)	3.1 (±0.3)	3.5 (±0.6)
	*Ile	5.1 (±0.3)	4.7 (±0.3)	4.9 (±0.1)	4.9 (±0.3)
	*Leu	8.1 (±0.2)	8.0 (±0.3)	8.5 (±0.1)	8.2 (±0.3)
	*Lys	6.2 (±0.4)	6.0 (±0.5)	5.8 (±0.2)	6.0 (±0.3)
	*Met	1.1 (±0.5)	1.9 (±0.6)	1.6 (±0.0)	1.5 (±0.5)
	*Phe	4.2 (±0.4)	3.9 (±0.4)	3.7 (±0.1)	3.9 (±0.3)
	*Thr	3.9 (±0.2)	4.0 (±0.2)	3.8 (±0.0)	3.9 (±0.1)
	*Trp	1.0 (±0.1)	0.9 (±0.1)	0.8 (±0.0)	0.9 (±0.1)
	*Val	6.8 (±0.3)	6.1 (±0.3)	6.8 (±0.1)	6.6 (±0.5)
	*Cys	0.9 (±0.2)	1.0 (±0.2)	0.8 (±0.1)	0.9 (±0.1)
	*Tyr	7.8 (±0.5)	5.9 (±0.3)	5.8 (±0.1)	6.5 (±1.1)
	Ala	7.3 (±0.3)	8.0 (±0.8)	10.5 (±0.3)	8.6 (±1.7)
	Arg	6.3 (±0.3)	8.1 (±0.2)	7.3 (±0.2)	7.2 (±0.9)
	Asx (Asn+Asp)	8.1 (±0.3)	9.3 (±0.8)	7.8 (±0.1)	8.4 (±0.8)
	Glx (Gln+Glu)	11.3 (±0.7)	11.8 (±0.5)	10.9 (±0.4)	11.3 (±0.6)
	Gly	5.0 (±0.2)	4.9 (±0.2)	5.8 (±0.2)	5.2 (±0.5)
	Pro	7.3 (±0.3)	6.6 (±0.4)	7.6 (±0.9)	7.2 (±0.6)
	Ser	5.3 (±0.6)	5.8 (±0.7)	4.4 (±0.2)	5.2 (±0.8)
Degree of amidation^d^					
(Asn+Gln)_[mol]_/ (Asx+Glx)_[mol]_		50 ± 4%	65 ± 5%	73 ± 3%	63 ± 12%
Nutrients [g/100 g insect, dwb]:^b^					
True protein (Σ[AAR*_*i*_*])		50.9 (±1.2)^a^	54.9 (±0.8)^b^	46.6 (±0.6)^c^	50.8 (±4.2)
Chitin		4.3 (±0.3)^a^	4.4 (±0.7)^a^	5.1 (±0.3)^a^	4.6 (±0.5)
Glycogen^c^		2.7 (±0.7)^a^	0.9 (±0.4)^b^	1.7 (±0.1)^b^	1.8 (±0.9)
Total nitrogen (*N_*total*_*)		9.42 (±0.30)^a^	10.45 (±0.16)^b^	8.74 (±0.03)^c^	9.54 (±0.87)
Amide nitrogen (*N*_amide_)^d^		0.56 (±0.04)^a^	0.87 (±0.08)^b^	0.72 (±0.03)^c^	0.72 (±0.16)
Pure protein conversion factor *k_*A*_*		5.75 (±0.07)^a^	5.51 (±0.10)^b^	5.49 (±0.09)^b^	5.58 (±0.16)
Nitrogen-to-protein conversion factor *k_*p*_*		5.41 (±0.08)^a^	5.25 (±0.12)^b, c^	5.33 (±0.07)^a, c^	5.33 (±0.10)

a Asterisk (*) denotes essential and semi-essential amino acids. Average values from samples of different commercial breeders: number of breeders were 3, 2, and 1 for mealworms, crickets, and locusts, respectively. See Tables S1–S3 in the Supplementary Material for the values of each breeder, as well as the moisture contents of the fresh weight.

b *Different letters denote significant differences (p < 0.05) between the insect species, with n = 16 for mealworms, n = 10 for crickets, and n = 4 for locusts*.

c *Detected as glucose by HPAEC-PAD after complete hydrolysis*.

d *Determined by liberating NH_3_ from amide side chains of Gln and Asn by the method of Mossé et al. (13) to obtain the amount of amide nitrogen (N_amide_) and to determine degree of amidation*.

The large variability of true protein contents for the two insect species in the literature could be caused either by different diets, growth conditions, or harvesting stages on one hand (28, 34), or be an analytical problem of differing protein recoveries on the other hand (7). Protein contents determined from the sum of AA residues always carry the risk of underestimating the actual protein content due to incomplete hydrolysis of the protein or degradation of AA. Labor-intensive control experiments to check the extent of actual protein recovery (18) are seldomly carried out in publications that include compositional analysis of insects, or are at least not explicitly mentioned. Potential underestimation of true protein is probably the reason why up until recently, many reports in the literature relied solely on crude protein when presenting insect protein contents, even when complete AA profiles were conducted for the same publication (27, 30–33). To avoid reporting true protein contents and conversion factors determined from AA data with unknown recoveries, several precautions were taken in this study to ensure accurate results for AA profiles and true protein contents:

- As is common, an internal standard (Norleu) was added to each sample before the 6 M HCl hydrolysis. Its determined recovery was used to correct for losses during the whole sample preparation (pipetting errors, AA degradation during hydrolysis), which improves both precision and accuracy;
- an in-house reference standard (mealworm batch M3a) was added to each sequence batch of analyses to monitor and confirm reproducibility (implemented for all types of analyses);
- the hydrolysis time was checked in preliminary experiments on the in-house reference standard to maximize AA recovery (compromise between minimizing incomplete hydrolysis on one hand, and AA degradation due to prolonged hydrolysis times on the other hand);
- pure bovine serum albumin (BSA) was used as reference protein to check the accuracy of AA quantification, and correction factors determined where appropriate, as well as BSA spiked in insect samples to identify matrix effects that could diminish the protein recovery.

The hydrolysis time of 24 h was compared to 48 h. While for the total protein content, there was no significant effect on time for all three tested insects (on average protein content ratio of 24 h/48 h = 0.99 ± 0.02), the sulfur AA Cys and Met exhibited extensive degradations with the higher hydrolysis time. The contents decreased by two thirds for Met and by a quarter for Cys due to the additional 24 h in the 48 h hydrolysis time point. Hence, 24 h was used as hydrolysis time for all experiments. These steps lead to excellent protein recoveries of >95% for both pure BSA and BSA in spiked insect samples (for method, see section Hydrolysis Time Pre-Tests and BSA-Spiking). The AA profiles, true protein contents, and conversion factors presented here should therefore be an accurate representation of insect composition of our samples from various breeders in Europe. For *locusta migratoria*, no directly or indirectly reported value could be found for true protein in the literature. This makes our study with 46.6 g/100 g the first published true protein content for adult locusts, as well as the first published complete AA profile (Table 1)^1^.

The chitin content was around 4–5 g/100 g (dwb; Table 1) for all three analyzed species and therefore in the lower range compared to literature values, which are between 4 and 9 g/100 g (10, 35–37). The used analytical method showed next to chitin's monomer glucosamine also a glucose peak in the ion exchange chromatogram, which originates from glycogen. The observed glycogen contents showed significant variabilities between the insect species and were in the range of 0.9–2.7 g/100 g (dwb). However, these low amounts of digestible carbohydrates in insects are nutritionally of low relevance.

The amino acid (AA) profiles between proteins of the three insect species was very similar, especially for the essential amino acids (Table 1; see Figure S4 for the graphical representation). Alanine (Ala), as well as asparagine+aspartic acid (Asx = Asn+Asp) and arginine (Arg) showed the highest variabilities. As they are non–essential AA, these variations are nutritionally not important. The observed AA profiles are comparable to profiles in the literature (1, 10, 27, 30, 38, 39).

**Figure 1:**
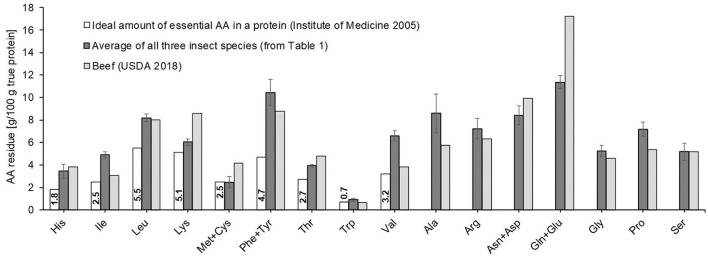
Amino acid (AA) profiles of insects (average from Table 1) and beef (40) for comparison, as well as the recommended composition of essential and semi-essential amino acids of a reference protein for children ≥1 year (41).

The average AA composition of the three edible insect species differed somewhat from the AA profile of dried beef, most importantly in the limiting AA when comparing to a reference protein with optimal AA profile based on the requirement for humans ≥1 year (Figure 1) (41). The sulfur-containing AA methionine + cysteine (Met+Cys) were with 2.0 ± 0.5 and 2.4 ± 0.1 g/100 g protein the lowest and hence limiting AA for mealworms and locusts, respectively, and in case of mealworms, significantly below the recommended level of 2.5 g/100 g in the reference protein (*p* = 0.028). This is corroborated by the literature, with sulfur-containing AA being the limiting component for most insect proteins (28). Crickets, on the other hand, fulfilled with 2.9 ± 0.6 g/100 g protein the recommended amount of Cys+Met. In comparison, the limiting AA for beef protein is tryptophan, which is with 0.65 g/100 g proteins slightly below the value of 0.7 g for the reference protein (40).

#### Samples From Different Breeders

**Figure 2:**
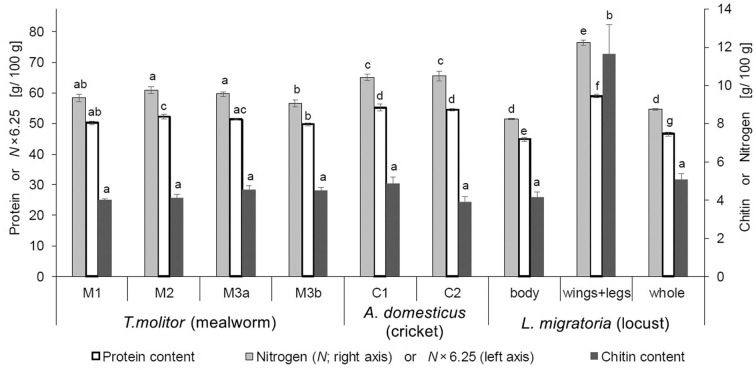
Average true protein (*n* = 3–6), chitin (*n* = 4) and nitrogen (*n* = 4) contents (dwb) of mealworm (*T. molitor*), cricket (*A. domesticus*), and locust (*L. migratoria*) samples from different breeders. Primary y-axis (left) is scaled to be exactly 6.25 times the secondary y-axis (right). Therefore, the total nitrogen content (right axis) also represents the crude protein content 6.25×*N* (left axis), which makes its apparent overestimation visible when compared to true protein (for exact values, see Table S1). Letters denote statistically different groups.

The chitin content for all analyzed samples was in the range of 4–5 g/100 g insect (dwb) and therefore no significant differences between breeders (M1 vs. M2 vs. M3; C1 vs. C2), and between batches from the same breeder (M3a vs. M3b) were observed (Figure 2). Only the chitin content of locust wings+legs, which were analyzed separately, was with 11.7 ± 1.5 g/100 g (dwb) significantly higher and more than double the level of the locust main body part. Insect wings+legs mainly consist of exoskeleton and must be highly resistant against external forces. Consequently, not only chitin, but also protein contents are higher in these body parts to increase the strength and mechanical properties of the exoskeleton by binding to chitin, making sclerotized body parts rich in protein (33, 42–44). Wings+legs represent around 10–15% of the whole locust weight (dwb), which can lead to a significantly higher total protein content of +~2 g/100 g (dwb), if the whole insect is processed compared to only the main body part (*p* = 0.008). However, the extremities are often removed for human consumption because wings and legs, especially of locusts, are stiff and not pleasant to chew. The small gain in nutritional value through inclusion of locust limbs (e.g., in powder form to make it palatable) due to the higher total protein content is probably mitigated by the impaired digestibility due to the concomitant higher chitin levels (45). The AA profiles, however, are virtually the same when comparing locust body with the whole locust, despite some significant differences in AA profiles between locust body vs. locust wings+legs (see Table S2). Wings+legs making up (for our samples) only 12.7% (dwb) of the whole locust insect is the reason for no significant differences between AA compositions of whole locusts vs. edible locust body. This is fortunate, as AA profiles of insects are usually reported for the whole insect in the literature, but these profiles may still be used when only interested in the palatable part of locusts.

The true protein contents of dried mealworms and dried crickets of different breeders were all within a close range around 50 and 55 g/100 g, respectively (Figure 2). Additionally, the AA composition of mealworm and cricket samples from different breeders were very similar (see Table S2). This is in accordance with proteins having specific functions throughout the whole insect body to maintain body functions, and therefore, AA profiles (meaning the individual AA amounts relative to each other as a fraction of the total protein) are similar between insects of a specific species regardless of the breeder and diet, whereas variations in the total protein content may still occur (27).

### Nitrogen-to-Protein Conversion Factor *k_*p*_*

The *k*_*p*_(= Σ[AAR_*i*_]/*N*_*total*_) of mealworms, crickets and locusts were very similar and on average with 5.33 (±0.10) significantly lower than Jones' default 6.25 factor (*p* < 0.0001; see Table 1). It is evident that use of 6.25 × *N*_*total*_, which is still widely applied, significantly overestimates protein contents for the three tested insects by on average 17%. The strategy sometimes applied to correct for non-protein nitrogen in insects when using *k*_*p*_ = 6.25, namely to first subtract the calculated nitrogen portion originating from quantified chitin [6.25 × (*N*_*total*_–*N*_*chitin*_)], does with still ~13% overestimation little to improve the situation. The amino acid profile or other non-protein nitrogen components seem to be the main responsible factor, while chitin is only responsible to a quarter for the discrepancy between the actual *k*_*p*_ and Jones' default 6.25 for pure proteins.

Janssen et al. (10) determined a *k*_*p*_ of 4.75 for mealworms, which is in agreement with Belghit et al.'s (11) reported values between 4.64 and 4.86. These two reports are to the best of our knowledge the only directly reported literature values for mealworms, and are on average ~12% smaller compared to the findings of our study (*k*_*p*_ = 5.41 ± 0.08; Table 1). Interestingly, this correlates with their ~12% smaller average true protein content compared to ours (average ~44 vs. our 51 g/100 g dwb). With *k*_*p*_ being a function of true protein and *N*_*total*_, this indicates that *N*_*total*_ must be virtually the same as ours, which is indeed the case for Janssen et al. (9.4 g/100 g dwb; Belghit et al. did not report *N*_*total*_, and crude protein contents only on wet basis). The twice as high chitin content observed by Janssen et al. compared to our average content for mealworms (9 vs. 4.3 ± 0.3 g/100 g dwb) only explains a quarter of the 12% discrepancy in *k*_*p*_ and true protein compared to our values, leaving ~9% relative difference originating as discussed above (section Protein, Nitrogen and Chitin Composition) from differences in the sample itself (rearing conditions, e.g., diet, development/molting stage at the time of harvest), from differences in actual protein recovery due to different analytical methods, or arguably most likely, a combination thereof. For crickets, the only conversion factors directly reported in the literature of 4.53 and 4.80 (11) correspond to 9–14% smaller values compared to our average *k*_*p*_ of 5.25 ± 0.12 (Table 1), and hence exhibit virtually the same tendencies of lower literature *k*_*p*_ as for mealworm larvae.

While the discussed papers of Janssen et al. (10) and Belghit et al. (11) have the only directly reported conversion factors for any of the three insect species relevant to this study, *k*_*p*_ may be calculated from other literature sources from published crude protein recoveries (in %), defined in the literature as true protein (sum of anhydrous AA residues, Σ[AAR_*i*_]) divided by crude protein (6.25 × *N*_*total*_), hence *k*_*p*_ = (crude protein recovery [%]) × 6.25.^2^ For mealworm larvae, calculated *k*_*p*_ from the studies of Finke (27, 32) and Yi et al. (39) are with 5.3–5.7 considerably higher than Janssen et al.'s (10) and Belghit et al.'s (11) factors (4.6–4.9), but in excellent agreement with our average result of 5.41±0.08 from in total four mealworm batches of three different breeders (Table 1). For crickets, conversion factors between 5.02 and 5.66 can be calculated from the data of Nakagaki et al. (38), Finke (27), and Yi et al. (39), and are again in the same range as our average *k*_*p*_ = 5.25±0.12 from two different cricket breeders. In fact, when calculating conversion factors from data of a review by Xiaoming et al. (46) using the reported average crude and true protein contents of 9 insect orders (*Ephemeroptera, Odonata, Orthoptera, Homoptera, Hemiptera, Coleoptera, Magaloptera, Lepidoptera, Hymenoptera*) from up to 100 different insect species, the resulting average *k*_*p*_ of 5.4 ± 0.5 is in excellent agreement with the 5.33 ± 0.12 average of this study (see Table S4 in the Supplementary Material for the calculation of *k*_*p*_ from extracted literature data). Hence, the default 6.25 × *N*_*total*_ is not appropriate for edible insects, despite its universal application. The insect-specific conversion factor of 5.33 determined in this study as an average from 7 different batches of 3 insect species reflects a more accurate total protein content and is widely supported by data extracted from the literature, even for insect species of other orders.

### Amide Nitrogen, Nitrogen Distribution, and Conversion Factor *k_*A*_*

The other conversion factor of practical relevance is the pure protein conversion factor *k*_*A*_, which is calculated using the protein nitrogen content (*N*_*protein*_). The larger the abundance of AA containing high amounts of nitrogen, for example arginine or histidine, the lower the conversion factor will be (47). The exact nitrogen content of insect protein, however, cannot be determined directly after complete hydrolysis, as it does not allow distinguishing between some AA with different numbers of nitrogen atoms, namely Asn vs. Asp and Gln vs. Glu, since all amide side chains in Asn and Gln are hydrolyzed to the corresponding carboxylic acids in Asp and Glu (R-CONH_2_ + H_2_O → R-COOH + NH_3_). This makes no difference for the total protein content, but a significant difference for the *N*-distribution and *k*_*A*_. Hence, the amide nitrogen (*N*_*amide*_), which corresponds to the molar amounts of Asn+Gln in the protein, had to be quantified separately, and we chose the method of Mossé et al. (13) based on a milder hydrolysis (2 M HCl, 3 h at 115°C), followed by quantification of released NH_3_. This contrasts to “total NH_3_” quantified after total protein hydrolysis through 6 M HCl which is sometimes reported in AA tables (27), but is known to poorly represent Asn+Gln amide nitrogen especially in insects, as chitin also releases significant amounts of NH_3_ during the applied much harsher hydrolysis conditions (33). Hence, our separately determined *N*_*amide*_ contents of 0.56, 0.87, and 0.72 g/100 g (dwb) translate to degrees of amidation (Gln+Asn)/(Glx+Asx) of 50, 65, and 73% for mealworm larvae, crickets, and locusts, respectively (see Table 1), and represent to the best of our knowledge the first reported amide values for insects in the literature.

**Figure 3:**
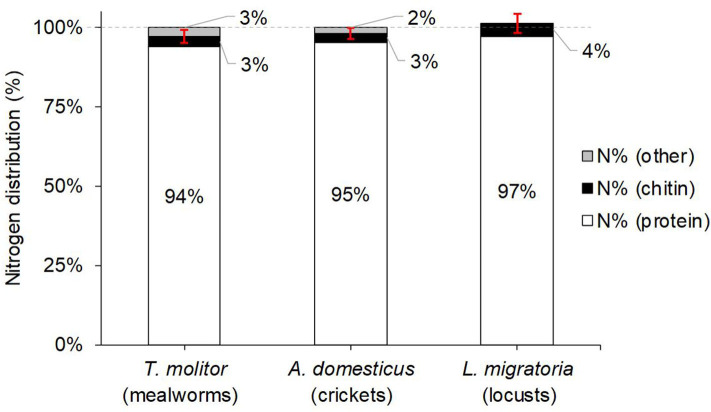
Average relative nitrogen (*N*) distribution of the studied edible insect species, coming from protein, chitin, or other sources. Protein nitrogen was calculated from the nitrogen contents of the detected amino acid residues + the separately determined amide nitrogen content (from Asn and Gln side chains; see Equation (3) in section Total Nitrogen and Amide Nitrogen Contents). The red error bars indicate the 95% confidence interval for N%(protein)+N%(chitin). N%(other) was calculated from 100%–N%(protein)–N%(chitin). Note that for locusts, N%(protein)+N%(chitin) exceeds with 101% the 100% mark slightly, but is within the margin of error.

The determined *N*_*amide*_ contents allowed calculation of *N*_*protein*_, and hence determination of relative nitrogen distribution N% between protein, chitin, and other sources [with N%(other) = 100% – N%(protein) – N%(chitin); see Figure 3]. It is evident that only 0–3% of total nitrogen is unidentified, whereas most nitrogen (94–97%) is originating from the insect protein. For mealworms and crickets, the unidentified portion of 2–3% is small, but statistically significant ≠ 0% (*p* = 0.010 and 0.038, respectively), indicating the presence of other nitrogen compounds than protein or chitin. For locusts, on the other hand, N%(protein)+N%(chitin) is not significantly different from N%(total) (101 ± 1% vs. 100%, respectively; *p* = 0.2204). Compared to the available literature, our N%(protein) of 94% for mealworms was again—as was the case for true protein content and *k*_*p*_—well above the values from Janssen et al. (10) of 77–88%.

With *N*_*protein*_, we were able to calculate *k*_*A*_ = *true protein*/ *N*_*protein*_, and observed a close range of values for all three studied insects between 5.49 and 5.75 (Table 1). The average *k*_*A*_ of 5.58 ± 0.13 was very close to but significantly different from our average *k*_*p*_ of 5.33 ± 0.10 (*p* = 0.0012). The relative difference of 5% between the two factors correlates with on average 5% non-protein nitrogen observed in the three insect species. Mathematically, *k*_*A*_ = *k*_*p*_ if no non-protein nitrogen species are present, as *N*_*total*_ = *N*_*protein*_. Hence, *k*_*A*_ calculated from AA profiles of whole insects can be compared to literature *k*_*p*_ values determined from insect protein isolates (assuming *N*_*other*_ ≈ 0). Indeed, our *k*_*A*_ of 5.75 ± 0.07 for mealworms (Table 1) is in agreement with the conversion factor calculated for a mealworm protein extract of 5.59 by Janssen et al. (10). Hence, *k*_*A*_ is mostly applicable if no other nitrogen sources are present apart from protein, e.g., for calculating the protein content from the nitrogen content of protein isolates.

## Conclusion

In order to study the protein quality and establish specific nitrogen-to-protein conversion factors for edible insects sold on the Swiss market, batches of *T. molitor* (mealworm larvae), *A. domesticus* (house crickets), and *L. migratoria* (locusts) were obtained from three, two, and one commercial European breeders, respectively, and thoroughly analyzed for complete amino acid (AA) profiles, as well as accurate true protein, nitrogen, and chitin contents.

This study has confirmed that protein contents in insects have been significantly overestimated in literature and in industry alike due to use of 6.25 × *N*_*total*_ as crude protein, and that chitin nitrogen subtraction before crude protein calculation is not a sufficient correction. In the West, protein content has been among the main arguments to contemplate consumption of edible insects, making a correct representation a crucial factor. Conclusively, the conversion factor *k*_*p*_ = 5.33 of this study, which was determined as an average from 7 different batches of 3 insect species, reflects a more accurate true protein content than 6.25 × *N*_*total*_, and is widely supported by data extracted from the literature, even for insect species of other orders.

Separate determination of amide nitrogen allowed for the first time the calculation of precise protein nitrogen (*N*_*protein*_) contents and the degree of amidation for insects, which was in the range of 50–73%, giving a more complete picture of the AA profiles of the 3 insect species with regard to Asn+Gln vs. Asp+Glu. It also allows for calculating the nitrogen distribution and the pure protein conversion factor *k*_*A*_. Protein nitrogen turned out to be on average 95% of total nitrogen (*N*_*total*_), which explains why the average *k*_*A*_ defined as *true protein*/*N*_*protein*_ is with 5.6 only 5% larger compared to our already mentioned average *k*_*p*_ = *true protein*/*N*_*total*_ = 5.33.

Although true protein contents are significantly below the widely reported crude protein contents, insect true proteins are with on average 51 g/100 g (dwb) still relatively high, and together with high levels of other nutrients as well as their sustainable breeding possibilities, insects retain their promising potential to satisfy the growing meat demand by replacing animal proteins. Nevertheless, it is the consumer's decision to accept edible insects as food in order to make use of this great potential—a decision that should be made on the basis of correctly reported protein contents, e.g., by using our average *k*_*p*_ of 5.33 for whole insects, and *k*_*A*_ = 5.6 for insect protein isolates.

## Data Availability Statement

All datasets generated for this study are included in the article/Supplementary Material.

## Author Contributions

LN and SB conceived the idea and designed the work. AT performed the experiments. AT and SB performed the data analysis and interpretation. All authors contributed to the manuscript and approved the final version.

## Conflict of Interest

The authors declare that the research was conducted in the absence of any commercial or financial relationships that could be construed as a potential conflict of interest.

## Footnotes

^1^Osimani et al. (30) published AA profiles without Cys and Trp (and Val and Met reported as the sum Val+Met), whereas Bednárová et al. (25) analyzed locust nymphs, not adult locusts.

^2^There is an inconsistency in reporting AA contents in the literature, either as mass/mass ratios of (1) the detected whole free AA (taking up one H_2_O during hydrolysis of the peptide chain; “uncorrected”), or (2) the anhydrous AA residues (AAR = AA – H_2_O) that directly represent the amounts in the protein polymer chain (as reported in this study; “corrected”). For the following calculations from the literature, it was checked if anhydrous AAR or AA were reported, and corrections applied where necessary (eg., sum of reported amino acids ≥ crude protein for whole insects strongly indicates uncorrected values). For example, the study of Finke (32) needed corrections, even though Finke (27) reported both uncorrected and corrected crude protein recoveries.
