# Supplementary material for: Nitrogen-to-Protein Conversion Factors for Edible Insects on the Swiss Market: T. molitor, A. domesticus, and L. migratoria
Source: Front Nutr. 2020 Jul 10;7:89. doi: 10.3389/fnut.2020.00089 (PMC7366252; doi:10.3389/fnut.2020.00089)
Supplement: Supplementary file 1 [file Data_Sheet_1.pdf]

Supplementary Material

Nitrogen-to-Protein Conversion Factors for Edible Insects on the Swiss Market: *T. molitor*, *A. domesticus*, and *L. migratoria*

Samy Boulos, Anina Tännler and Laura Nyström*

Laboratory of Food Biochemistry, Institute of Food, Nutrition and Health, Department of Health Sciences and Technology, ETH Zurich, 8092 Zurich, Switzerland.

*** Correspondence:** Laura Nyström: laura.nystroem@hest.ethz.ch

Supplementary Equations, Tables & Figures

- Equations for error propagation Page 2
- Tables S1–S5 Page 3
- Figures S1–S3 Page 7

Equations for error propagation

Standard deviations of averages calculated from other averages that already have a standard deviation (e.g. averaged AA composition of the three insect species) were calculated taking the quadrature of the internal (SD_{int}) and external standard deviation (SD_{ext}) (see Eq. (S1)).

(S1) $$SD_{tot} = \sqrt{SD_{int}^2 + SD_{ext}^2}$$

SD_{int} is calculated according to Eq. (S2) from the equally weighted standard deviations of the individual averages (SD_i), with n = number of averages being averaged (e.g. for the average of the three insect species, $n = 3$). SD_{ext} is calculated as a usual standard deviation from the individual averages x_i and the overall average $\bar{x}$ (Eq. (S3)).

(S2) $$SD_{int} = \sqrt{\sum_{i=1}^{n} \left(\frac{1}{n} SD_i\right)^2}$$

(S3) $$SD_{ext} = \sqrt{\frac{\sum_{i=1}^{n} (x_i - \bar{x})^2}{n-1}}$$

Standard deviations of quantities derived from multiplication or division of measured averages are calculated according to classical error propagation (assuming no correlation of errors) (Ku 1966). E.g. for $k_p = protein/N$, the standard deviation would be:

(S4) $$SD_{k_p} = k_p \sqrt{\left(\frac{SD_{Protein}}{Protein}\right)^2 + \left(\frac{SD_N}{N}\right)^2}$$

Standard deviations of quantities derived from addition or subtraction of measured averages are calculated according to classical error propagation (assuming no correlation of errors) (Ku 1966). E.g. for $N_{protein+chitin} = N_{protein} + N_{chitin}$,[1] the standard deviation would be:

(S5) $$SD_{N_{protein+chitin}} = \sqrt{\left(SD_{N_{protein}}\right)^2 + \left(SD_{N_{chitin}}\right)^2}$$

Reference

Ku, H.H. (1966). Notes on the Use of Propagation of Error Formulas, *J. Research of National Bureau of Standards-C. Engineering and Instrumentation* 70C(4): 263–273. doi:10.6028/jres.070c.025.

[1] E.g. calculated to compare with N_{total} for significant differences to determine if other nitrogen species (N_{other}) are relevant.

Tables S1–S5

Table S1: Total chitin, nitrogen, true protein content (all on dwb), and protein overestimation [%] when using the generally used 6.25×N_{total} for blanched insect samples from different breeders (illustrated in **Figure 2**).

Insect species	Batch/ breeder	Chitin [g/100 g insect]	Total nitrogen [g/100 g insect]	True protein [g/100 g insect]	Overestimated protein content [%][a]
T. molitor (mealworms)	M1	4.0 (±0.1)	9.4 (±0.2)	50.4 (±0.6)	16.0 (±0.4)
	M2	4.1 (±0.2)	9.7 (±0.2)	52.3 (±0.8)	16.3 (±0.4)
	M3a	4.6 (±0.2)	9.6 (±0.1)	51.3 (±0.2)	16.3 (±0.2)
	M3b	4.5 (±0.2)	9.1 (±0.2)	49.7 (±0.4)	13.8 (±0.3)
A. domesticus (crickets)	C1	4.9 (±0.4)	10.4 (±0.2)	55.2 (±1.1)	18.0 (±0.5)
	C2	3.9 (±0.3)	10.5 (±0.3)	54.3 (±0.3)	20.3 (±0.5)
L. migratoria[b] (locusts)	body	4.4 (±0.3)	8.2 (±0.03)	44.8 (±0.7)	14.8 (±0.2)
	wings+ legs	11.7 (±1.5)	12.2 (±0.1)	58.9 (±0.6)	29.8 (±0.4)
	whole	5.1 (±0.3)	8.7 (±0.03)	46.6 (±0.6)	17.1 (±0.2)

[a] Overestimation is calculated as (crude protein/true protein) – 100% = ((6.25×N_{total})/Σ[AAR_i]) – 100%.

[b] Locust main body weight fraction (dwb) was 87.3%, whereas wings+legs were the remaining 12.7%. Whole locust data was calculated by weighted average of the body×87.3% + (wings+legs)×12.7%.

Table S2: Amino acid (AA) composition [g/100 g true protein] of blanched mealworms (*T. molitor*), crickets (*A. domesticus*) and locusts (*L. migratoria*) from different batches/ breeders.[a]

AA residue [g/100 g true protein]	*T. molitor* (mealworms)				*A. domesticus* (crickets)		*L. migratoria* (locusts)[b]		
	M1	M2	M3a	M3b	C1	C2	body	wings+legs	whole
*His	4.35 (±0.07)	3.83 (±0.28)	3.94 (±0.10)	4.49 (±0.07)	3.04 (±0.27)	3.15 (±0.40)	3.07 (±0.39)	3.57 (±0.28)	3.13 (±0.34)
*Ile	5.07 (±0.09)	4.89 (±0.44)	4.99 (±0.19)	5.46 (±0.05)	4.56 (±0.40)	4.77 (±0.44)	4.96 (±0.15)	4.65 (±0.56)	4.92 (±0.15)
*Leu	8.19 (±0.07)	7.90 (±0.39)	7.89 (±0.18)	8.23 (±0.09)	7.90 (±0.35)	8.02 (±0.53)	8.52 (±0.03)	8.63 (±0.32)	8.53 (±0.05)
*Lys	6.41 (±0.25)	5.89 (±0.82)	6.10 (±0.48)	6.61 (±0.04)	6.09 (±0.63)	5.98 (±0.67)	6.11 (±0.21)	3.92 (±0.65)	5.83 (±0.2)
*Met	0.67 (±0.07)	1.26 (±0.42)	1.76 (±0.35)	0.83 (±0.05)	2.05 (±0.73)	1.82 (±0.83)	1.70 (±0.03)	0.77 (±0.23)	1.58 (±0.04)
*Phe	4.58 (±0.04)	3.84 (±0.63)	4.04 (±0.34)	4.29 (±0.02)	3.76 (±0.55)	4.00 (±0.59)	3.90 (±0.06)	2.31 (±0.51)	3.70 (±0.08)
*Thr	3.72 (±0.07)	4.04 (±0.30)	4.13 (±0.15)	3.89 (±0.32)	3.99 (±0.25)	4.08 (±0.24)	3.95 (±0.05)	3.11 (±0.04)	3.85 (±0.04)
*Trp	0.97 (±0.02)	1.07 (±0.04)	1.09 (±0.01)	1.05 (±0.01)	0.85 (±0.06)	0.95 (±0.02)	0.83 (±0.03)	0.32 (±0.02)	0.76 (±0.02)
*Val	6.96 (±0.10)	6.53 (±0.46)	6.60 (±0.24)	7.16 (±0.07)	6.11 (±0.43)	6.04 (±0.34)	6.56 (±0.13)	8.57 (±0.58)	6.81 (±0.14)
*Cys	0.72 (±0.03)	0.82 (±0.13)	1.09 (±0.30)	0.91 (±0.05)	1.01 (±0.29)	0.97 (±0.17)	0.79 (±0.10)	0.97 (±0.04)	0.81 (±0.08)
*Tyr	7.74 (±0.07)	7.12 (±0.36)	8.05 (±0.20)	8.27 (±0.06)	6.15 (±0.21)	5.75 (±0.28)	5.65 (±0.11)	6.96 (±0.02)	5.82 (±0.09)
Ala	7.42 (±0.04)	7.56 (±0.14)	7.18 (±0.20)	6.94 (±0.11)	8.53 (±0.30)	7.40 (±0.28)	9.76 (±0.29)	15.7 (±0.74)	10.51 (±0.27)
Arg	6.04 (±0.03)	6.64 (±0.09)	6.17 (±0.10)	6.30 (±0.27)	7.99 (±0.07)	8.19 (±0.18)	7.38 (±0.17)	6.42 (±0.36)	7.26 (±0.15)
Asx (Asn+Asp)	7.98 (±0.10)	8.09 (±0.99)	8.23 (±0.27)	8.09 (±0.09)	8.82 (±0.57)	9.78 (±0.64)	8.13 (±0.09)	5.68 (±0.27)	7.82 (±0.08)
Glx (Gln+Glu)	10.75 (±0.38)	12.16 (±0.99)	11.28 (±0.26)	10.99 (±0.56)	11.87 (±0.68)	11.82 (±0.59)	11.35 (±0.49)	7.69 (±0.49)	10.89 (±0.43)
Gly	5.27 (±0.06)	5.06 (±0.24)	4.88 (±0.12)	4.96 (±0.07)	4.98 (±0.10)	4.79 (±0.19)	5.63 (±0.18)	6.85 (±0.07)	5.78 (±0.15)
Pro	7.61 (±0.17)	7.47 (±0.54)	7.23 (±0.12)	6.93 (±0.39)	6.82 (±0.54)	6.37 (±0.34)	7.40 (±1.00)	9.31 (±0.37)	7.61 (±0.90)
Ser	5.55 (±0.04)	5.83 (±0.74)	5.36 (±0.32)	4.62 (±0.17)	5.47 (±0.78)	6.09 (±0.67)	4.36 (±0.20)	4.60 (±1.10)	4.40 (±0.23)
Degree of amidation[c]	51 ± 3%	49 ± 4%	45 ± 3%	53 ± 5%	63 ± 6%	68 ± 5%	70 ± 3%	103 ± 6%	73 ± 3%

[a] Asterisk (*) denotes essential and semi-essential amino acids.

[b] For locusts, extremities (wings+legs) were analyzed separately from the main body part, and Whole locust data was calculated by weighted average of the body×87.3% + (wings+legs)×12.7%.

[c] Degree of amidation [%] = $(Asn+Gln)_{[mol]}$ / $(Asx+Glx)_{[mol]}$ × 100%.

Table S3: Moisture & dry matter content [g/100 g fresh weight] of blanched insect samples.[a]

Insect species	Breeder/ batch	Moisture content [g/100 g insect, fresh weight]	Dry matter content [g/100 g insect, fresh weight]
T. molitor (mealworms)	M1	57.8	42.2
	M2	68.9	31.1
	M3a	71.2	28.8
	M3b	72.0	28.0
A. domesticus (crickets)	C1	70.4	29.6
	C2	70.8	29.2
L. migratoria (locusts)	Whole insect	65.1	34.9

[a] Calculated from single batch weight changes during freeze drying, while taking into account the additional moisture content of the freeze-dried powders to get the total moisture and dry matter contents reported here. Hence, no standard deviation of the total moisture and dry matter contents can be given.

Table S4: Calculated protein recoveries & conversion factors k_p from literature crude protein ($6.25\times N_{total}$) and true protein data (sum of AA residues) of 9 insect orders taken from Table 1 in the review of Xiaoming *et al*. (2010). Overall mean k_p is reported with ± its standard deviation.[a]

Order	Crude Protein (= $6.25\times N$)	Total AA residues ($\Sigma(AAR_i)$)	Protein recovery (= $\Sigma(AAR_i)$/ crude protein)	k_p (= recovery×6.25)
**Ephemeroptera**	66.26	65.97	99.6%	6.22
**Odonata**	58.83	46.03	78%	4.89
Isoptera		44.03		
**Orthoptera**	44.1	38.87	88%	5.51
**Homoptera**	51.13	42.45	83%	5.19
**Hemiptera**	55.14	48.72	88%	5.52
**Coleoptera**	50.41	39.74	79%	4.93
**Magaloptera**	56.56	53.31	94%	5.89
**Lepidoptera**	44.91	32.88	73%	4.58
Diptera	59.39			
**Hymenoptera**	47.81	45.18	94%	5.91
dry weight basis [%]			**Mean Protein recovery** **86%**	**mean k_p** **5.40 (± 0.55)**

[a] Source: Text and numbers in black is data (average values) from Table 1 in review of Xiaoming, C., Ying, F., Hong, Z. and Zhiyong, C. (2010). "Review of the nutritive value of edible insects" in "Forest insects as food: humans bite back", *Proceedings of a workshop on Asia-Pacific resources and their potential for development*, pages 85-92. Text in blue indicates values calculated using the literature data.

Table S5: Separate analysis of protein, chitin, nitrogen, and conversion factors for locust main body and locust wings+legs, as well as the values for the whole insect calculated from the weighted averages.

**Composition** [g/100 g insect, dwb]	Locust body [87.3% (w/w)]	Locust wings+legs [12.7% (w/w)]	Locust whole [100% (w/w)]
Protein	44.82 (±0.67)	58.94 (±0.57)	46.61 (±0.59)
Chitin	4.14 (±0.27)	11.65 (±1.52)	5.09 (±0.31)
Total nitrogen (N_{total})	8.23 (±0.03)	12.24 (±0.14)	8.74 (±0.03)
Amide nitrogen (N_{amide})	0.69 (±0.03)	0.92 (±0.03)	0.72 (±0.03)
Protein nitrogen ($N_{protein}$)	8.14 (±0.11)	10.94 (±0.21)	8.50 (±0.10)
Degree of amidation [%]	70% (±3%)	103% (±6%)	73% (±3%)
$(N_{protein} + N_{chitin})/N_{total}$ [%]	102%	96%	101% (±1%)
$\boldsymbol{k_A}$	5.50 (±0.11)	5.39 (±0.12)	5.49 (±0.09)
$\boldsymbol{k_p}$	**5.45 (±0.08)**	**4.81 (±0.07)**	**5.33 (±0.07)**

Figures S1–S3

(A)

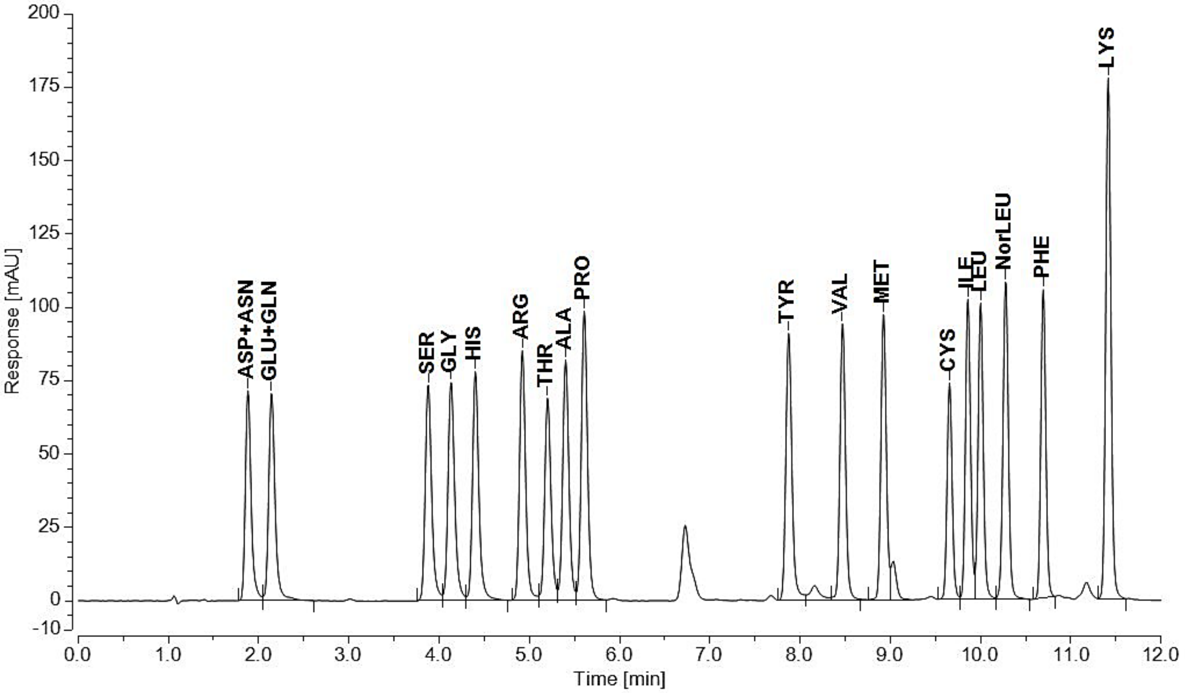

(B)

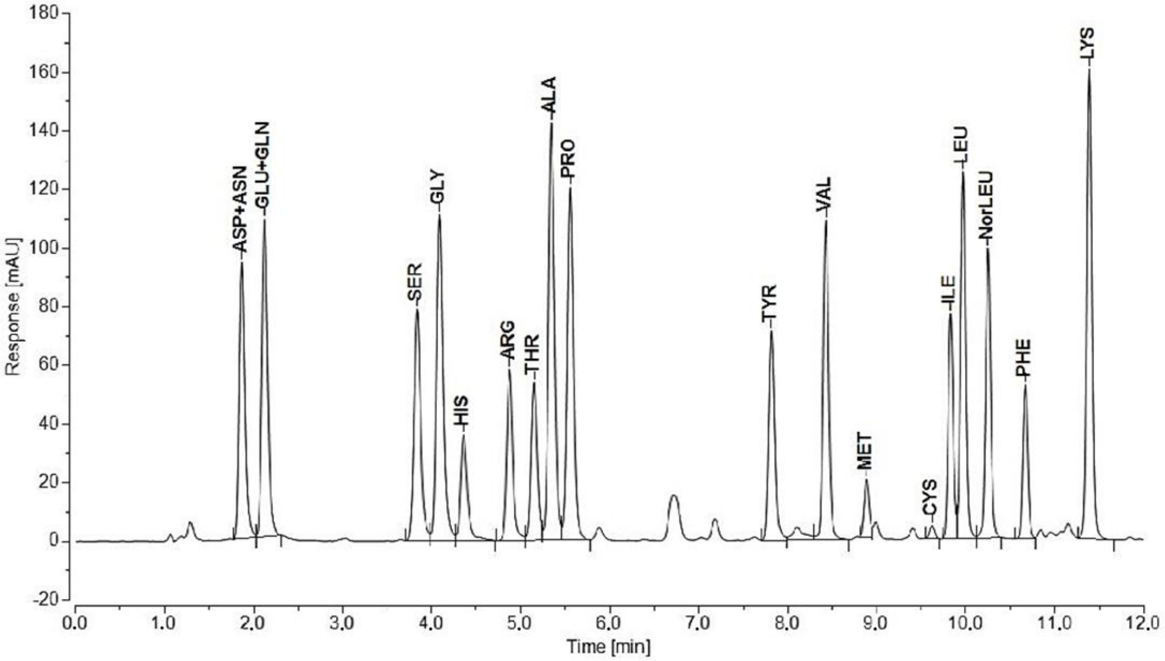

Figure S1: Chromatogram of (A) standards and (B) mealworm sample showing separated AA by RP-HPLC-DAD after acid hydrolysis with 6 M HCl and PITC derivatization. See Materials and Methods for details.

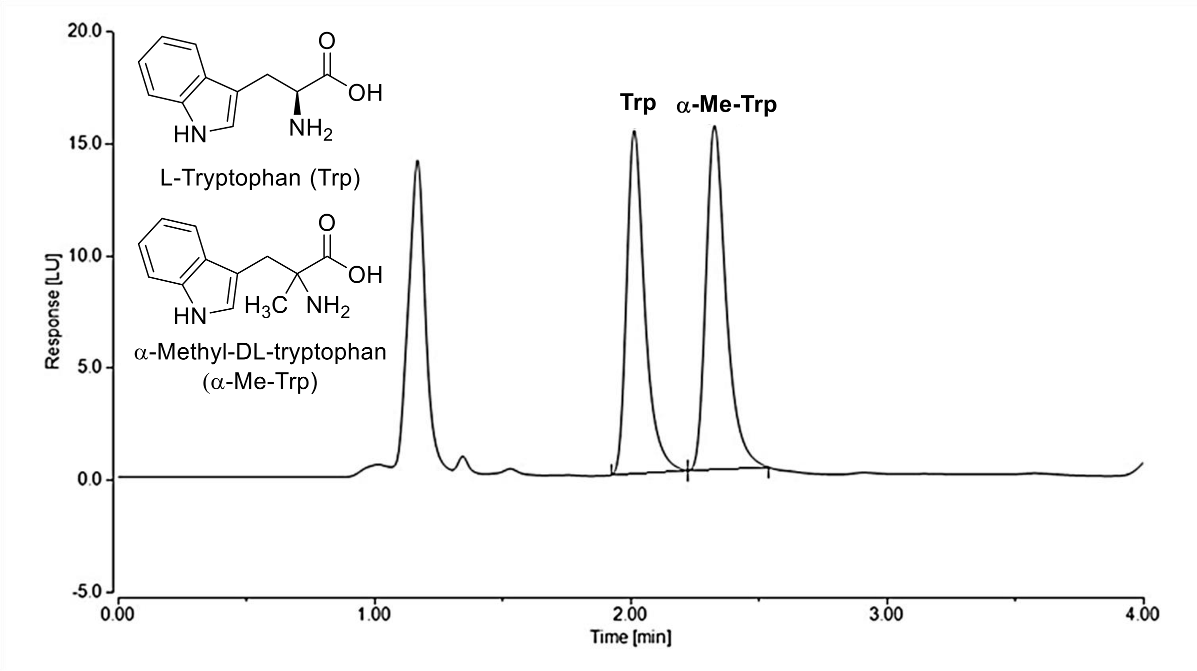

Figure S2. RP-HPLC-FLD chromatogram of a mealworm sample after hydrolysis with NaOH to release tryptophan (Trp; with α-methyl-DL-tryptophan as internal standard). See Materials and Methods for details.

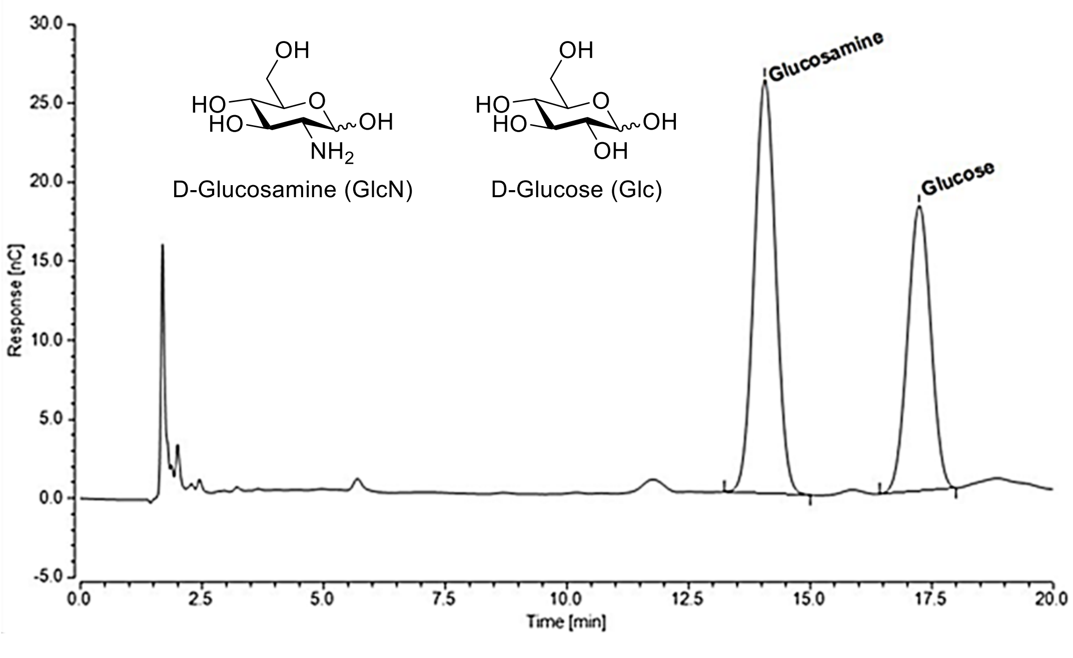

Figure S3. HPAEC-PAD chromatogram of a mealworm sample showing sugar monomers GlcN and Glc after hydrolysis with H_2SO_4 for the quantification of chitin and glycogen, respectively. See Materials and Methods for details.
